# Alteration of Neural Pathways and Its Implications in Alzheimer’s Disease

**DOI:** 10.3390/biomedicines10040845

**Published:** 2022-04-04

**Authors:** Sujin Kim, Yunkwon Nam, Hyeon soo Kim, Haram Jung, Seong Gak Jeon, Sang Bum Hong, Minho Moon

**Affiliations:** 1 Department of Biochemistry, College of Medicine, Konyang University, 158, Gwanjeodong-ro, Seo-gu, Daejeon 35365, Korea; aktnfl3371@naver.com (S.K.); yunkwonnam@gmail.com (Y.N.); sooya1105@naver.com (H.s.K.); gkfkawjd@gmail.com (H.J.); jsg7394@naver.com (S.G.J.); harryhong0314@gmail.com (S.B.H.); 2 Research Institute for Dementia Science, Konyang University, 158, Gwanjeodong-ro, Seo-gu, Daejeon 35365, Korea

**Keywords:** Alzheimer’s disease, neural pathways, neural circuits, neurodegeneration, connectome

## Abstract

Alzheimer’s disease (AD) is a neurodegenerative disease accompanied by cognitive and behavioral symptoms. These AD-related manifestations result from the alteration of neural circuitry by aggregated forms of amyloid-β (Aβ) and hyperphosphorylated tau, which are neurotoxic. From a neuroscience perspective, identifying neural circuits that integrate various inputs and outputs to determine behaviors can provide insight into the principles of behavior. Therefore, it is crucial to understand the alterations in the neural circuits associated with AD-related behavioral and psychological symptoms. Interestingly, it is well known that the alteration of neural circuitry is prominent in the brains of patients with AD. Here, we selected specific regions in the AD brain that are associated with AD-related behavioral and psychological symptoms, and reviewed studies of healthy and altered efferent pathways to the target regions. Moreover, we propose that specific neural circuits that are altered in the AD brain can be potential targets for AD treatment. Furthermore, we provide therapeutic implications for targeting neuronal circuits through various therapeutic approaches and the appropriate timing of treatment for AD.

## 1. Introduction

Alzheimer’s disease (AD) is the most common type of dementia in the elderly and is a significant health problem worldwide. The incidence of AD increases with age, with nearly 35% of 85-year-olds suffering from AD [1,2]. AD has been characterized by the aggregation and accumulation of amyloid-β (Aβ) and hyperphosphorylated tau. In addition, AD is a progressive neurodegenerative disease in which cognitive impairment is the main symptom [3]. Moreover, the secondary symptoms are as follows: psychiatric, sensory, and motor dysfunctions [4,5]. Therefore, AD treatment should inhibit cognitive decline and induce various clinical symptoms [6]. Based on many theories and hypotheses, many clinical trials are underway to treat AD. The U.S. Food and Drug Administration has recently approved an Aβ-binding monoclonal antibody, aducanumab (Aduhelm), through an accelerated approval pathway [7]. Although aducanumab may be beneficial in reducing in amyloid plaques in the brains of AD patients, it is not associated with behavioral improvement. Therefore, definitive treatment for cognitive and behavioral deficits is required.

Neural circuits are the main mediators of various behaviors controlled by the brain, from simple functions to complex cognitive processes [8]. Interestingly, it is known that Aβ and tau have been progressively impaired the synapses, neuronal circuits, and neural networks in the brain with AD [9,10]. Several studies using neural tracing and radiological imaging, such as diffusion tensor imaging (DTI) and functional magnetic resonance imaging (fMRI) have shown that neural pathways are altered in AD brains [11,12,13]. Damage to neural circuits in the AD brain can result in cognitive impairment, such as memory deterioration [14]. In addition, it has been suggested that the alterations in neural circuits and network function due to pathological changes in synaptic plasticity might be associated with several clinical symptoms, such as sensory and motor dysfunctions [4]. Interestingly, clinical studies have shown changes in the sensory systems of patients with early-stage AD, and that these changes take precedence over cognitive impairment [15,16,17]. From this perspective, neural circuitry could be a target for treating various symptoms of AD. Therefore, we selected the brain regions involved in changes in cognition and several clinical manifestations of AD, and summarized the alteration of efferent pathways from these AD-associated brain regions.

The challenge for neuroscience is to visualize the essential structural elements of the brain from the perspective of neural connections related to behaviors [12,18]. For the visualization of the neural connectivity or network related to behavior, there are three levels of brain connectivity: macroscale, mesoscale, microscale [19]. The macroscale connectome anatomically represents inter-area connections between distinct brain regions and shows the most large-scale connection patterns in the brain. In particular, DTI and fMRI are widely used to infer structural and functional connections in the living brain [20]. The microscale connectome represents the level of pre-and post-synaptic connections between single neurons. Microscale studies use electron and light microscopy to demonstrate neural connections at the ultrastructural level [21]. The mesoscale connectome represents intercellular connections between different neurons across different brain regions. In addition, mesoscale connectivity provides a detailed understanding of the cell-type composition of different brain regions, and the patterns of inputs and outputs that each cell type receives and forms, respectively [22]. Therefore, the mesoscale connectome can connect information collected at the level of both macroscale and microscale connectivity. In addition, at the mesoscale level, both long-range and local connections can be described using a sampling approach with diverse neuroanatomical tracers that enable whole-brain mapping in a reasonable time frame across many animals [23].

To understand AD-related cognitive, behavioral, and psychological symptoms, a precise connection of neural circuits should be examined. Moreover, understanding the alterations of neural circuits in the brain with AD might provide a better understanding of possible treatments that substantially affect the progression of AD. This review encompasses and summarizes recent discoveries in terms of impairments/alterations of neural circuits in the AD brain. In addition, we discuss various approaches for treating impaired neural circuits in the brain with AD.

## 2. Neural Circuits Associated with AD

### 2.1. Hippocampal Pathways

#### 2.1.1. Hippocampal Pathways in Healthy Brains

**Figure 1:**
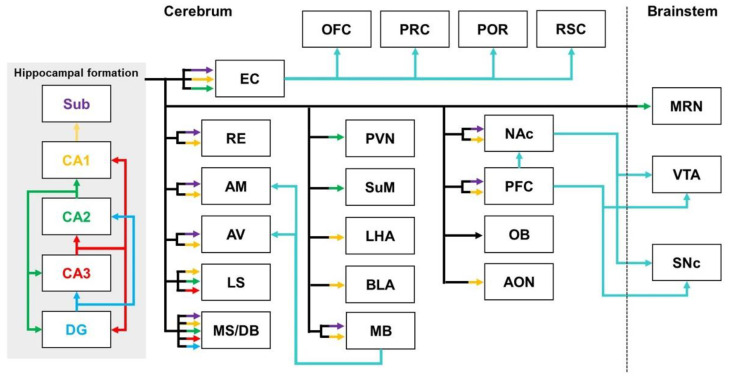
Schematic diagram of the hippocampal efferent pathways indicating projection patterns for the target regions: the yellow arrows represent the outputs from CA1; the green arrows indicate the outputs from CA2; the red arrows indicate the outputs from CA3; the purple arrows indicate the subiculum output; and the solid cyan line shows higher-order pathways. Abbreviations: AM—anteromedial thalamic nucleus; AON—anterior olfactory nucleus; AV—anteroventral thalamic nucleus; BLA—basolateral amygdala; EC—entorhinal cortex; LHA—lateral hypothalamic area; OFC—orbitofrontal cortex; LS—lateral septal nuclei; MB—mammillary bodies; MS/DB—medial septum/diagonal band of Broca nuclei; MRN—median raphe nucleus; NAc—nucleus accumbens; PFC—prefrontal cortex; POR—postrhinal cortex; PRC—perirhinal cortex; PVN—paraventricular nucleus; RE—nucleus reuniens of the thalamus; RSC—retrosplenial cortex; SNc—substantia nigra pars compacta; Sub—subiculum; SuM—supramammillary nucleus; VTA—ventral tegmental area.

The hippocampal formation, which includes CA1-3, the dentate gyrus (DG), and the subiculum, is one of the major regions of the limbic system [24]. The roles of the hippocampal formation are well established in cognitive functions, such as learning and memory [25,26]. In addition, hippocampal formation has been implicated in various behaviors, such as pain and social behavior [27,28]. The hippocampal formation constitutes a close connection between subregions and sends projections to various regions of the brain (Figure 1).

The close connections between the subregions of the hippocampal formation are well known. The hippocampal CA1 receives input from CA2 and CA3, and projects mainly to the subiculum [26]. Most projections from CA1/the subiculum connect to higher-order cortical regions via the entorhinal cortex (EC) [29]. CA2 sends projections to all hippocampal CA subregions, the DG, and the entorhinal cortex [30]. In contrast to CA1, which mainly connects to the deep layer of the entorhinal cortex, the projections from CA2 mostly innervate layer II of the entorhinal cortex [31]. CA3 is known to send projections to CA1, CA2, and the DG [30,32].

The hippocampal formation sends output projections to various regions, in addition to the subregions within the hippocampal formation. The hippocampal formation connects with cortical areas through several direct or indirect pathways [33]. The hippocampal–cortical pathway, through the medial/lateral entorhinal cortex, reaches the postrhinal, perirhinal, orbitofrontal, and retrosplenial cortices [34]. The hippocampal CA1/subiculum is also known to be directly connected to the medial/lateral prefrontal cortex (PFC) [35,36,37]. Furthermore, the PFC, receiving glutamatergic projections from CA1/the subiculum, sends γ-aminobutyric acid (GABA)ergic projections to various regions, including the core of the NAc, subthalamic nucleus, VTA, and SNc [38]. Hippocampal formation is also connected to the septal area. All hippocampal CA subregions have been reported to directly output to the septal area [39,40]. Furthermore, the DG and subiculum also directly innervate the medial septum/diagonal band of Broca nuclei (MS/DB) [40]. In addition to the areas mentioned above, the hippocampus also sends projections to the amygdala, thalamus, and hypothalamus. The connectivity of ventral CA1 with the basolateral amygdala is the one of the hippocampo-amygdala pathways [41]. The hippocampal formation sends projections to the anterior thalamic nucleus (ATN), which consists of the anteromedial, anterodorsal, and anteroventral nuclei [42,43]. The anteroventral thalamic nucleus is the target region of dorsal CA1/subiculum innervation [44]. In contrast to dorsal CA1, the output of ventral CA1/the subiculum reaches the anteromedial and anteroventral nucleus via the medial mammillary body of the hypothalamus [44]. Ventral CA1 projects to the lateral hypothalamic area (LHA) [45,46]. Hippocampal CA2 projections are sent to the paraventricular nuclei and supramammillary nucleus of the hypothalamus [39]. In addition, it has been proposed that some CA1/subiculum neurons interconnect with the nucleus reuniens of the thalamus (RE) and constitute the nucleus reuniens–CA1 circuit [47]. Hippocampal formation also projects to the mammillary nuclei, which are a part of the hypothalamus [48]. CA1/the subiculum innervate the medial mammillary nucleus pars lateralis, and medial mammillary nucleus pars medialis, of the mammillary body [49]. Neural connections from the hippocampal formation to the mammillary body lead to ATN through the mammillothalamic tract [43,50]. Hippocampal CA1/the subiculum indirectly modulate the ventral pallidum, ventral tegmental area (VTA), and substantia nigra pars compacta (SNc) via glutamatergic projections to the nucleus accumbens (NAc) shell [38]. In addition, the ventral subiculum has been reported to send direct glutamatergic projections to the NAc core [51]. Moreover, the hippocampus is related to the olfactory network, and it has been shown that the dorsoventral region of the hippocampus projects to the olfactory bulb (OB) and anterior olfactory nucleus (AON) [12,52]. It was confirmed that the AON received unidirectional synaptic input from CA1 of the hippocampus, unlike other regions that indirectly received input from the hippocampus [53]. Furthermore, the median raphe nuclei of the midbrain have also been reported as a target region for hippocampal CA2 innervation [39].

#### 2.1.2. Hippocampal Pathways in the Brains with AD

By examining the frontal hippocampal connectivity of patients with probable AD, it was demonstrated that the connectivity between the right frontal cortex and hippocampus was significantly reduced [54]. In addition, patients with early AD showed impairment of functional connectivity between the right hippocampus and various regions, such as the medial prefrontal cortex (PFC); ventral anterior and posterior cingulate cortex; right inferotemporal cortex; right cuneus; left cuneus; and the right superior and middle temporal gyrus [55]. Specifically, hippocampal diffusivity in early AD for hippocampal output regions, such as the intrahippocampal region, parahippocampal gyrus, and posterior cingulate cortex, was negatively correlated with 18F-fluorodeoxyglucose uptake [56]. Furthermore, patients with amnestic mild cognitive impairment (MCI) and AD showed decreased gray matter volume, decreased functional connectivity between the bilateral hippocampus and the region of interest, and impaired integrity of the fornix body [57]. Overall, the functional connectivity of the right hippocampus was significantly decreased in the early stages of AD, and the functional connectivity of the bilateral hippocampus reduced as the disease progressed. Moreover, decreased tyrosine hydroxylase innervation in the subiculum of a Tg2576 mouse model of AD caused a decrease in glutamatergic transmission from the dorsal subiculum to the core of the NAc [51]. Disruption of this connectivity can damage the VTA-hippocampus-NAc loop, which is involved in spatial memory, reward, and the formation of novelty and persistent memory, thus affecting memory loss and cognitive impairment in AD patients [58]. Another study providing topographical evidence between the hippocampus and the septum, using neural tracers in 5XFAD mouse models, reported that the hippocampo-septal pathway exhibited degeneration at both the early and late stages of AD [40]. In 4.5-month-old 5XFAD mice compared with WT mice, projections from the DG/subiculum to the MS were impaired, and 14-month-old 5XFAD mice had reduced projections leading to the MS in all subregions of hippocampal formation [40]. Furthermore, the functional associations of the long-range CA1 lateral septal nuclei (LS) inhibitory circuits have been shown to be reduced in the J20 mouse model of AD [59]. Altered spike-theta coordination and reduced phase-amplitude coupling between septal-theta and CA1-theta were observed in a young J20 group compared to a control group, suggesting damage to the circuitry between CA1 and the LS [59]. Moreover, dendritic loss of CA1 and basolateral amygdala neurons, and impairment of hippocampus- and amygdala-related memory, occured in APP/PS1 mice [60]. Given that hippocampo-amygdala interaction plays a vital role in contextual fear conditioning [61], it could be speculated that alteration of the hippocampo-amygdala pathway may be involved in the impairment of contextual fear conditioning in the AD model [62]. In addition, impairment of hippocampal pathways to the thalamus and mammillary body in AD can induce significant functional deficits. The hippocampo-mammillary body pathway is essential for normal memory function [50]. It has been suggested that the degeneration of projections from the subiculum and fornix to the mammillary body and ATN may contribute to episodic memory loss in AD [63,64,65]. Furthermore, impairment of the hippocampo-mammillary body pathway may be a key contributor to disorders of visuospatial orientation and memory [65]. Since the mammillary body and thalamus are major members of the Papez circuit and the hippocampus [66], circuit disconnection due to the impairment of hippocampal pathways can be fatal to cognitive function. Based on previous studies, we have summarized those impairments of the hippocampal pathway that occur in AD (Table 1). Unfortunately, few studies have directly examined the altered efferent pathways in animal models of AD. Further studies are needed to measure the impairment of individual hippocampal pathways and their neurobehavioral implications.

### 2.2. Septal Pathways

#### 2.2.1. Septal Pathways in Healthy Brains

**Figure 2:**
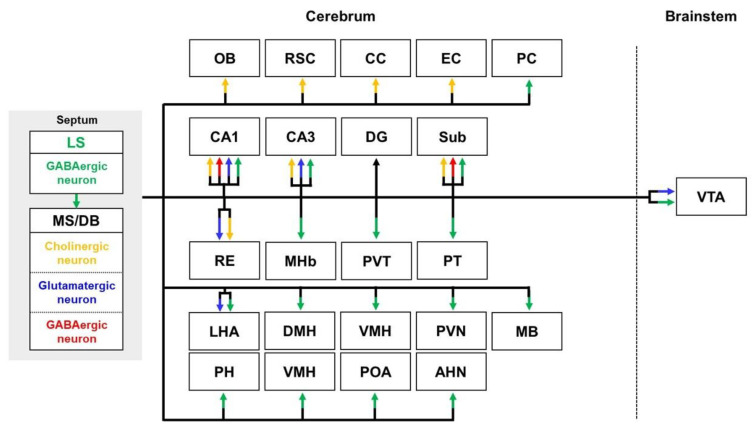
Schematic diagram of septal outputs indicating projection patterns from the LS and MS/DB to the target regions. The yellow, blue, and red arrows represent the cholinergic, glutamatergic, and GABAergic pathways from the MS/DB, respectively. Green arrows indicate LS projections. Abbreviations: AHN, anterior hypothalamic nucleus; CC, cingulate cortex; DG, dentate gyrus; DMH, dorsomedial hypothalamic nucleus; EC, entorhinal cortex; LHA, lateral hypothalamic area; LS, lateral septal nuclei; MB, mammillary body; MHb, medial habenula; MS/DB, medial septum/diagonal band of Broca nuclei; OB, olfactory bulb; PC, piriform cortex; PH, posterior hypothalamic nucleus; POA, preoptic area; PT, paratenial nucleus; PVH, paraventricular hypothalamic nucleus; PVT, paraventricular thalamus; RE, nucleus reuniens; RSC, retrosplenial cortex; Sub, subiculum; VMH, ventromedial hypothalamus; VTA, ventral tegmental area.

The septal area is composed of two major regions: the lateral septal nuclei (LS) and MS/DB [72,73] (Figure 2). The LS are a brain region characterized by an abundance of GABAergic neurons [74] and are well known for taking input from the hippocampal formation and sending outputs to the MS/DB [73,75]. In addition, the LS innervate the hippocampus; hypothalamus; thalamus; midbrain; and CA3 of the ventral hippocampus and its adjacent regions, the subiculum and the piriform cortex [76,77]. Efferent projections of the LS reach most of the hypothalamus, such as the median, medial, and lateral preoptic area; anterior, posterior, dorsomedial, ventromedial, and paraventricular hypothalamic nucleus; LHA; and mammillary body [77,78,79,80,81]. Moreover, the LS sends its output to parts of the thalamus, such as the medial habenula, paraventricular thalamus, paratenial nucleus, and nucleus reuniens [77]. The LS also projects to the VTA of the midbrain [77,82].

There are three cell types in MS/DB projection to the hippocampus: cholinergic, glutamatergic, and γ-aminobutyric acid GABAergic neurons [83,84]. The cholinergic MS/DB-hippocampal pathway sends to hippocampal CA1 and CA3 [85,86]. The MS/DB sends GABAergic and cholinergic projections to the subiculum and CA1 of the hippocampus [87]. The glutamatergic MS/DB-hippocampal pathway innervates hippocampal CA1 and CA3 [88]. Although the association between the MS/DB and DG is known, there are no studies on which types of neurons/projections are associated with reciprocal connectivity between the MS/DB and DG. In addition, it is known that the MS/DB sends cholinergic projections to various regions, such as the entorhinal cortex, cingulate cortex, retrosplenial cortex, OB, and PFC [89,90,91,92]. The MS/DB modulates mesolimbic dopaminergic neurons by sending glutamatergic projections to the VTA of the midbrain [93]. The glutamatergic MS/DB projection also reaches the LHA of the hypothalamus [94]. The MS/DB is also projected onto the paratenial nucleus of the thalamus [78,95].

#### 2.2.2. Septal Pathways in the Brain with AD

Several studies have demonstrated that the connection between the MS and the hippocampus is impaired in the AD brain (Table 1). In particular, innervation from the MS to the hippocampus decreased by approximately 52% in 5XFAD mice compared to WT mice [12]. Another study revealed that the septo-hippocampal pathway began to degenerate in 4.5-month-old Aβ-overexpressing transgenic mice before neuronal loss in the MS [40]. In particular, interconnections between the MS and DG/subiculum and innervations from MS to CA3 were significantly impaired in 5XFAD mice before the onset of cognitive dysfunction. In addition, disruption of the interconnections with MS and hippocampal formation is accelerated, along with AD progression. A THY-Tau22 transgenic mouse, which developed tau pathology in both the hippocampus and the basal forebrain, showed disconnection between MS and the hippocampus [68]. Moreover, the connectivity of the MS and hippocampus in 16- to 18-month-old tauopathy mice was reduced compared to that in healthy mice and six- to eight-month-old tauopathy mice [67]. One study suggested that altered GABAergic septo-hippocampal pathways, together with functional deficits of phosphorylated tau–accumulating parvalbumin-positive neurons, were critical factors in cognitive decline and the alteration of hippocampal activity patterns present in the tau (VLW) mice [70]. In the hAPP-J20 mouse model of AD, a dramatic decrease in GABAergic septo-hippocampal innervation was seen in eight-month-old mice [69]. Furthermore, the loss of the GABAergic septo-hippocampal pathway resulted in the alteration of synchronous activity in the hippocampus of the AD brain [69]. In particular, the septal nucleus, which has strong reciprocal connectivity with the hippocampus, is a vulnerable area in AD. The reciprocal connectivity of the septal nucleus and hippocampus is called the septo-hippocampo-septal loop [72], and damage to this loop is well known in AD patients and animal models of AD [12,96]. Moreover, animals with damage in the MS and dorsal hippocampal formation mimic cognitive impairment in patients with AD [97]. Thus, impairment of the septum via AD-related pathologies could influence learning and cognitive disturbances [98], impairment of spatial and working memory [99,100], alteration of the hippocampal theta rhythm [101], anxiety-like behavior [102], and arousal [103]. Despite the potential clinical importance of the septal pathways in AD, the alteration of septal pathways other than the septo-hippocampal pathway is still not fully investigated. Furthermore, few studies have investigated the behavioral disorders of AD associated with the impairment of septal pathways. In further studies, whole efferents of the septum that are impaired in AD and their implications in AD-related behavioral disorders should be studied.

### 2.3. Locus Coeruleus Pathways

#### 2.3.1. Locus Coeruleus Pathways in Healthy Brains

**Figure 3:**
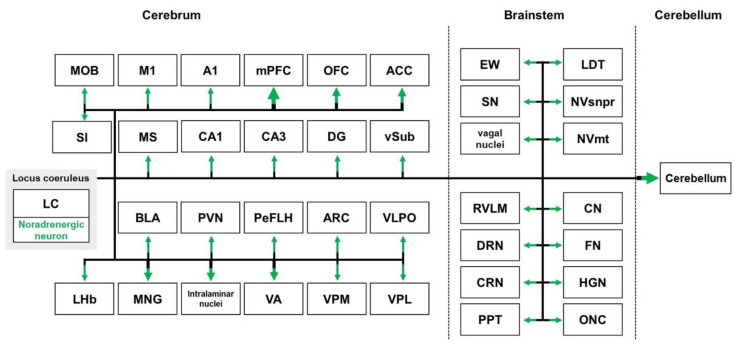
Schematic diagram of the noradrenergic (NA) pathway from the LC to various regions, such as the neocortex, pallidal regions, thalamus, hypothalamus, hippocampal formation, brainstem, and cerebellum. The thickness of the arrow indicates the degree of connectivity. A1—primary auditory cortex; ACC—anterior cingulate cortex; ARC—arcuate nucleus; BLA—basolateral amygdala; CN—cochlear nucleus; CRN—caudal raphe nuclei; DG—dentate gyrus; DRN—dorsal raphe nuclei; EW—Edinger–Westphal nucleus; FN—facial nucleus; HGN—hypoglossal nucleus; LDT—laterodorsal tegmental nuclei; LHb—lateral habenula; M1—primary motor cortex; MNG—midline nuclear group; MOB—main olfactory bulb; mPFC—medial prefrontal cortex; MS—medial septum; NVmt—trigeminal motor nucleus; NVsnpr—trigeminal sensory nucleus; OFC—orbitofrontal cortex; ONC—oculomotor nuclear complex; PeFLH—lateral hypothalamus/perifornical area; PPT—pedunculopontine tegmental nuclei; PVN—paraventricular nucleus; RVLM—rostral ventrolateral medulla; SI—substantia innominate; SN—salivatory nuclei; VA—ventral anterior nuclei; VLPO—ventrolateral preoptic area; VPL—ventral posterolateral nucleus; VPM—ventral posteromedial nucleus; vSub—ventral subiculum.

The locus coeruleus (LC), located in the lateral aspect of the fourth ventricle, is a major source of noradrenaline (NA) in the central nervous system (CNS) [104]. In the mammalian brain, axons of individual LC neurons branch extensively to innervate multiple brain regions of the neuraxis. The LC is well known for sending projections to various regions, such as the neocortex, limbic system, thalamus, hypothalamus, brainstem, and cerebellum [105,106] (Figure 3).

There are widespread projections of the LC to the cerebral cortex, which is the critical area for higher cognition, such as learning, memory, and affective function [107,108]. The cortical regions that receive projections from the LC are the medial PFC, orbitofrontal cortex, and anterior cingulate cortex (ACC) [109]. In particular, the medial PFC receives approximately half of the LC projections targeting the cerebral cortex [109]. In addition, the LC also plays important roles in sensory and motor functions through connection with the auditory cortex, main olfactory bulb (MOB), and motor cortex [110,111].

The LC is the major source of NA in the hippocampus, which is the most critical region for cognitive function [112]. Projections from the LC reach the hippocampal CA1, CA3, DG, and ventral subiculum [113,114]. Moreover, NA projections from the LC to the basolateral amygdala are important for stress responses, anxiety-like behavior, and aversive learning [115,116,117]. The LC also sends outputs to the substantia innominate and MS, which are part of the basal forebrain [118,119].

The thalamus and hypothalamus are important regions that link information from the LC to the cerebral cortex. The LC provides intense innervation to parts of the thalamus, especially to the intralaminar and midline nuclei, lateral habenula, and ventral anterior nuclei [120,121,122]. In addition, the LC exhibits sparse innervation to the somatosensory thalamus, such as the ventral posteromedial nucleus and ventral posterolateral nucleus of the thalamus [123,124]. It has also been reported that there is connectivity from the LC to the hypothalamus, including the ventrolateral preoptic area, paraventricular nucleus, lateral hypothalamus/perifornical area, and arcuate nucleus [105].

The LC contributes to several physiological responses and sensory and motor functions via outputs to the brainstem. NA axons originating from the LC reach the parasympathetic preganglionic nuclei, including the Edinger–Westphal nucleus, salivatory nuclei, and parasympathetic vagal nuclei [105]. The LC also connects with the dorsal raphe nuclei, pedunculopontine tegmental nuclei, and laterodorsal tegmental nuclei [125,126,127]. Some of the projections originating from the LC are sent to the motor nuclei, such as the facial nucleus, hypoglossal nucleus, trigeminal motor nucleus, and oculomotor nuclear complex [105]. In addition, the LC is associated with correct motor performance by sending powerful projections to the cerebellum [128].

#### 2.3.2. Locus Coeruleus Pathways in the Brains with AD

A previous study revealed that innervation from the LC to the hippocampus was decreased in 5XFAD mice [12]. Interestingly, the LC–hippocampal pathway was the most severely degenerated circuit in the Aβ-overexpressing brain. Thus, it can be speculated that the damage to the LC–hippocampal pathway by Aβ and tau is strongly attributable to cognitive impairment in AD. Several studies have suggested that loss of NA innervation can contribute to the initiation, progression, and severity of AD [129,130]. Surprisingly, the loss of NA neurons in the LC is up to 70% in AD brains [131,132]. In particular, the massive loss of LC-NA neurons in the brain with AD might result in the degeneration of efferent pathways from the LC to several target regions (Table 1). Cognitive impairment in AD may be caused by loss of the LC–medial PFC pathway, accounting for more than half of the LC projections to the target regions [133]. Early impairment of the olfactory and auditory systems may result from the loss of the LC–MOB and LC–primary auditory cortex (A1) pathways [110,134,135]. The disruption of NA projections from the LC to the basolateral amygdala, which makes an important contribution to anxiety-like behavior and aversive learning, may occur in AD patients with anxiety [115,116,117]. Concomitant circadian rhythm disturbances in AD can be associated with impairment of the pathway from the LC to GABAergic neurons in the ventrolateral preoptic area [136,137]. Although the LC pathway is important in AD pathogenesis, few studies have examined alterations in LC circuitry. Unfortunately, no studies have investigated whether changes in the neural circuits originating from LC can be directly associated with AD-related cognitive and behavioral dysfunction.

### 2.4. Substantia Nigral Pathways

#### 2.4.1. Substantia Nigral Pathways in Healthy Brains

**Figure 4:**
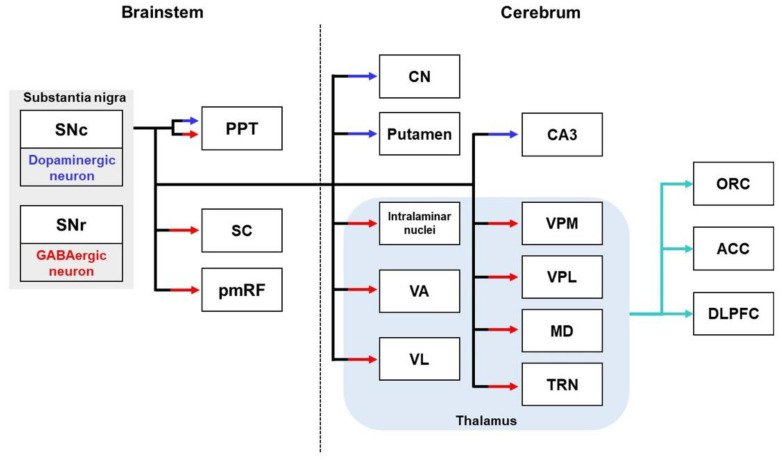
Schematic diagram showing each dopaminergic pathway and GABAergic pathway from the SN to the target regions. The substantia nigra pars compacta (SNc) sends dopaminergic projections to the striatum, hippocampus, and brainstem. The substantia nigra pars reticular (SNr) provides GABAergic projections to the thalamus and brainstem. The solid cyan line shows higher-order cortical pathways. ACC—anterior cingulate cortex; CN—caudate nucleus; DLPFC—Dorsolateral prefrontal cortex; MD—mediodorsal nucleus; ORC—orbitofrontal cortex; PF—parafascicular nucleus; pmRF—pontomedullary reticular formation; PPT—pedunculopontine tegmental nucleus; SC—superior colliculus; TRN—thalamic reticular nucleus; VPL—ventral posterolateral nucleus; VA—ventral anterior nuclei; VL—ventral lateral nucleus; VPM—ventral posteromedial nucleus.

The substantia nigra (SN) comprises midbrain dopaminergic nuclei [138]. The SN consists of two major regions: the substantia nigra pars compacta (SNc) and substantia nigra pars reticular (SNr) [138]. The SNc projects mostly dopaminergic axons, and the SNr projects mainly GABAergic axons [139] (Figure 4). The SNc sends a dopaminergic projection to the putamen and caudate nucleus of the dorsal striatum [140,141]. In addition, the SNc sends dopaminergic projections to the pedunculopontine tegmental nucleus (PPT) of the brainstem [142]. The SNr GABAergic neurons have three major target regions: the thalamus, superior colliculus (SC), and PPT [143,144]. SNr sends an inhibitory GABAergic projection toward the medial thalamus through the nigrothalamic pathway [145,146]. Neural tracing studies indicate that SNr neurons send projections to the thalamic nucleus, including the intralaminar nuclei, as well as the ventral posterolateral, ventral posteromedial, ventral anterior, ventral lateral, mediodorsal, and thalamic reticular nuclei [147,148,149,150,151]. These GABAergic nigrothalamic pathways are the largest tract of the SNr neurons, and the thalamus is the most prominent target region of SNr innervation [152,153,154]. The nigrothalamic pathway plays a crucial role in the basal-ganglia—cortical loop [155]. In this loop, the thalamic nuclei receiving GABAergic projections from the SNr send glutamatergic modulation to the cortical areas, including the dorsolateral prefrontal cortex, anterior cingulate cortex, and OFC [156,157]. The second main target of SNr neurons is the SC, which is known as the nigrocollicular pathway [158]. SNr neurons also innervate the PPT and pontomedullary reticular formation (pmRF) [144,159,160].

#### 2.4.2. Substantia Nigral Pathways in the Brains with AD

Several studies have reported deficits in the SN pathways in AD. Histological studies in AD transgenic mice have reported deficits in the nigrostriatal pathway [161] (Table 1). Consistently, imaging studies have reported impaired nigrostriatal pathways in the brains of patients with AD [162,163]. The mesostriatal pathway from the SNc to the caudate nuclei and putamen nuclei is also impaired in patients with AD [162]. Moreover, impairment of the nigro-hippocampal pathway was detected in Aβ-overexpressing transgenic mice using neural circuit tracing [12]. Considering that the dopaminergic system contributes to AD symptoms [58], impairment of the nigro-hippocampal pathway may be associated with impaired cognitive function in AD. Interestingly, in the postmortem brains of AD patients, the SN showed an accumulation of Aβ plaques and NFTs [164,165]. These Aβ plaques and NFTs result in neuronal loss in the SN, and may be related to the alteration of the neural circuit and clinical symptoms of AD. Unfortunately, few studies are investigating the alteration of substantia nigral pathways and their functions in the AD brain. Thus, whether impairment of the substantia nigral pathways might influence AD-related symptoms should be further studied.

### 2.5. Visual Pathways

#### 2.5.1. Visual Pathways in Healthy Brains

**Figure 5:**
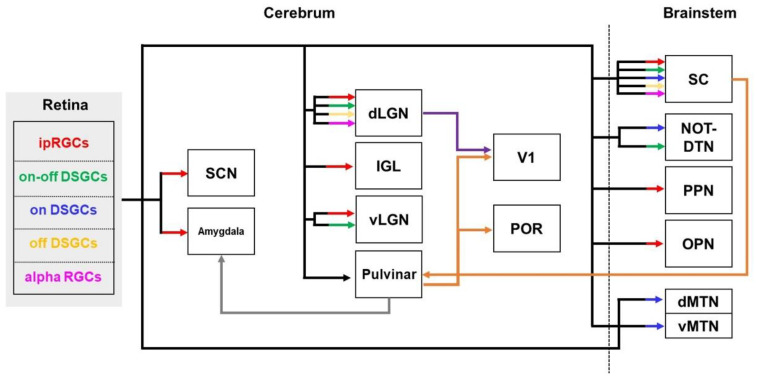
Schematic diagram of visual efferent pathways indicating projection patterns from the retina to brain regions: the solid black lines represent the pathways from the retina to the retinorecipient regions; the solid purple line indicates the geniculate pathway; and the solid orange line shows the colliculo-pulvinar pathway. dLGN—dorsal lateral geniculate nucleus; dMTN—dorsal medial terminal nucleus; DSGCs—direction-selective ganglion cells; IGL—intergeniculate leaflet; ipRGCs—intrinsically photosensitive retinal ganglion cells; NOT-DTN—nucleus of the optic tract-dorsal terminal nucleus; OPN—olivary pretectal nucleus; POR—postrhinal cortex; PPN—posterior pretectal nucleus; SC—superior colliculus; SCN—suprachiasmatic nucleus; vLGN—ventral lateral geniculate nucleus; vMTN—ventral medial terminal nucleus; V1—primary visual cortex.

The visual pathways constructed by the eye–brain connection induce various light-induced behaviors, such as conscious image forming and subconscious non-image forming visual functions [166,167]. Retinal ganglion cells (RGCs) project visual information to over 50 retinorecipient areas to provide image-forming functions, such as visual perception, and contribute to non-image-forming functions, such as the pupillary light reflex and circadian photoentrainment [168]. To date, the connectivity between the retina and retinorecipient areas and its roles are well known [168,169] (Figure 5).

The visual functions that form images can distinguish the shape, color, and movement of objects within the field of view [170]. The lateral geniculate nucleus and SC are the major brain regions related to image forming visual function [171,172]. The dorsal lateral geniculate nucleus (dLGN), a thalamic visual center, links the retina and visual cortical areas [173]. Additionally, the information from the retina into the dLGN is projected into the primary visual cortex (V1) through the classical visual pathway [174]. This efferent projection is called the retino-geniculo-cortical pathway [168,175]. The SC, which is one of the major areas contributing to sensorimotor transformations, receives inputs from most RGCs in the retina [174,176]. In addition, the SC sends projections to the higher-order cortical areas, such as the V1 and post-rhinal cortex (POR), through the pulvinar. The visual response of the POR is independent of V1, and the function of the POR is predominant in its ability to distinguish moving objects compared to V1. These connections are collectively called the retino-colliculo-pulvinar pathway [177].

The accessory optic system consists of the brainstem visual nuclei, such as the medial terminal nucleus (MTN) and nucleus of the optic tract (NOT)–dorsal terminal nucleus (DTN), which receives visual information directly from RGCs through accessory optic tracts. The connectivity between the RGCs and the brainstem visual nucleus contributes to slip-correction eye movement, which stabilizes the visual image and improves vision [178]. The MTN is connected with the RGCs that encode top–bottom motion, and this connectivity is associated with the behavior of vertical eye movements. The nucleus NOT–DTN receives projections from RGCs that encode forward motions and contribute to the function of horizontal eye movements [179].

Non-imaging visual regions, in which melanopsin-containing intrinsically photosensitive RGCs (ipRGCs) are primarily connected, are involved in the regulation of non-image-forming visual functions, such as the pupillary light reflex, circadian photoentrainment, sleep, and mood [180]. Information from ipRGCs is relayed to the suprachiasmatic nucleus (SCN), the vLGN, and the intergeniculate leaflet (IGL) areas, which are involved in circadian photoentrainment of the brain. In addition, ipRGCs send projections toward the olivary pretectal nucleus (OPN) and the posterior pretectal nucleus (PPN) regions, which are involved in the pupillary light reflex [181,182,183]. Moreover, a recent study identified GABAergic circuits between the retina and non-image-forming regions that weaken the sensitivity of non-image-forming functions [184].

#### 2.5.2. Visual Pathways in the Brains with AD

One study using neural tracers in an AD animal model reported that impairment of the retino-collicular pathway was observed in three-month-old 3xTg mice [71]. Another study using DTI in AD patients showed damage to visual pathways, including the optic nerves, optic tract, and corpus callosum [11]. Impairment of the pathway from the eye to the visual cortex may contribute to visual impairment in AD. In particular, patients with AD have been shown to exhibit abnormalities in visual functions, such as visual acuity, contrast sensitivity, color vision, motion and depth perception, and visual field, as well as difficulties in reading and finding objects [185,186]. In addition, defects of visual attention, visuospatial construction, and visual memory have also been observed in individuals with AD [187]. Moreover, deficits in image-forming functions and disturbances in non-image-forming functions, including the circadian rhythm and pupillary light reflex, are also present in the patients with AD [188,189].

Alterations in visual neural circuits are associated with AD-related pathologies in the retina [190,191]. In the early stage of AD, an accumulation of Aβ plaques and NFTs was observed in the retinas of mouse models of AD, and these abnormal depositions promoted inner retinal degeneration; this included a loss of the axons and dendritic spines of RGCs, as well as a reduction in the thickness of the retinal nerve fiber layer (RNFL) [71,192]. In patients with AD, histological analysis showed a 4.7-fold increase in retinal Aβ_42_ plaques compared to age-matched controls [193]. In addition, the aggregation of Aβ and tau in the retina of an AD mouse model increased the number of microglial cells [194]. In summary, damage to RGCs and their axons appears in the early stage of AD, suggesting an alteration in connectivity between the eye and the brain. Unfortunately, although the importance of visual function in AD is well known, only a few studies have investigated alterations in neural circuits associated with visual function (Table 1). Thus, future studies to reveal the altered visual pathways according to the stages of AD could provide crucial insights for its treatment.

### 2.6. Olfactory Pathways

#### 2.6.1. Olfactory Pathways in Healthy Brains

**Figure 6:**
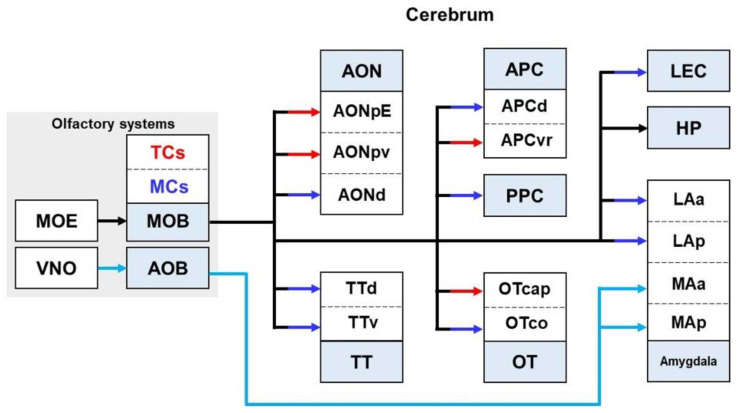
Schematic diagram of olfactory outputs indicating projection patterns from the olfactory bulb to the olfactory cortex. The MOB pathways, represented by solid black lines, are divided into parallel pathways originating from TCs and MCs: red arrows indicate the TC pathways; dark blue arrows indicate the MC pathways; and the light blue arrows indicate the AOB pathways. AOB—accessory olfactory bulb; AON—anterior olfactory nucleus; AONd—dorsal part; AONpE—pars external; AONpv—posterior ventral part; APC—anterior piriform cortex; APCd—dorsal part; APCvr—ventrorostral subdivision; HP—hippocampus; LAa—anterior lateral amygdala; Lap—posterior lateral amygdala; LEC—lateral entorhinal cortex; Maa—anterior medial amygdala; Map—posterior medial amygdala; MCs—mitral cells; MOB—main olfactory bulb; MOE—main olfactory epithelium; OT— olfactory tubercle; OTcap—cap part; OTco—cortical part; TCs—tufted cells; TT—tenia tecta; TTd—dorsal part; TTv—ventral part; VNO—vomeronasal organ.

In mammals, the olfactory system is divided into two distinct systems: the main and accessory olfactory systems. The main olfactory system consists of the MOB as the primary center and the main olfactory epithelium as the receptor [195] (Figure 6). In contrast, the accessory olfactory system consists of the accessory olfactory bulb (AOB) as the primary center and the vomeronasal organ (VNO) as receptors [196,197]. The main olfactory system detects airborne substances, whereas the accessory system senses fluid-phase stimuli. The olfactory information generated by odorous molecules in contact with olfactory receptor neurons is transmitted by olfactory pathways, leading to diverse behavioral and physiological functions such as neuroendocrine regulation, reproduction, social behavior, communication, food-finding, and selection [198,199].

Several studies have shown that the MOB pathway sends information to olfactory regions, such as the AON, tenia tecta (TT), olfactory tubercle (OT), piriform cortex (PC), amygdala, and lateral EC [200,201]. The MOB pathway projects into the olfactory cortex as a distinct pathway depending on both the mitral cells (MCs), which convey slow signals, and tufted cells (TCs), which convey fast signals [202,203]. Interestingly, axons from TCs mainly reach the anterior region of the olfactory region, whereas efferent projections of MCs project to most olfactory regions other than the TC projection regions [202]. The pathways from TCs send olfactory signals to the pars external and posterior ventral part of the AON, the ventrorostral subdivision of anterior PC, and the cap part of the OT. In addition, the target areas from the MCs include the dorsal and ventral TT, dorsal AON, cortical part of the OT, dorsal part of the anterior PC, posterior PC, anterior and posterior lateral amygdala, lateral olfactory tract, and lateral EC [202,204]. TCs induce olfactory behavior with a short-latency response when odor cues dissociate easily, whereas MCs have a longer latency than TCs to maintain behavioral accuracy through fine odor discrimination in a mixture of similar odors [202]. Moreover, a study investigating the molecular receptive range reported that MCs showed a strong inhibitory molecular receptive range, whereas TCs exhibited a weak or absent inhibitory molecular receptive range [184]. Likewise, unlike the MC pathway, the TC pathway has no concurrent inhibition and induces large single excitatory post-synaptic potentials [205].

In animal studies, it has been reported that innervation from the AOB mainly reaches the medial amygdala [206,207]. This AOB–amygdala pathway is important for sex-hormone-induced social behavior and reproductive behavior [208,209]. The functions of the AOB are well known in animals. However, in humans, VNO has regressed and AOB is non-existent [210,211]. Therefore, in this review, we only describe the structure and function of the MOB pathway, excluding AOB.

#### 2.6.2. Olfactory Pathways in the Brain with AD

**Figure 7:**
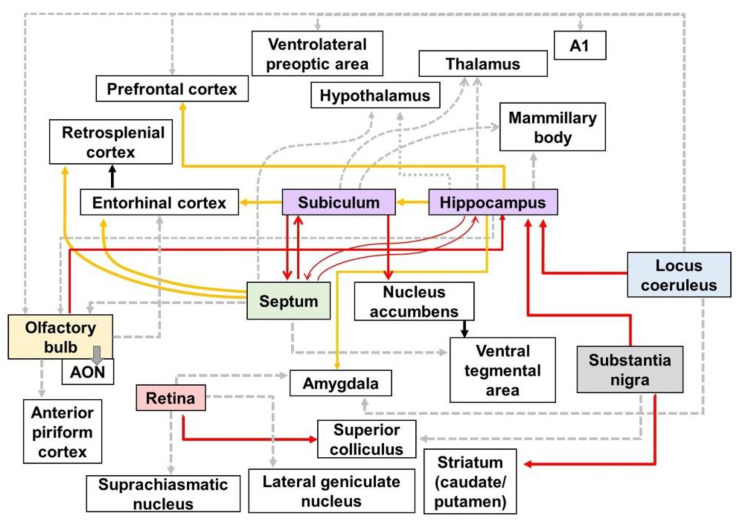
Schematic diagram of altered connections in the AD brain: Red, yellow, and gray lines directed away from the boxes represent efferent fibers which have synaptic contact with each other. The red lines show the altered efferent pathways in AD brain using the neural tracers identified in Table 1. The yellow lines indicate the output pathways that were investigated using various methods, such as electrophysiology, biomedical imaging technologies, and immunohistochemical stanning. The gray dotted lines represent the efferent pathways that affect various symptoms of AD, although the alteration in the connectivity of gray dotted lines has not been directly visualized. A1—primary auditory cortex; AON—anterior olfactory nucleus.

Impairment of the olfacto-hippocampal pathway has been detected in AD transgenic mice [12] (Table 1, Figure 7). The degeneration of the olfacto-hippocampal connection suggests that it may be the underlying mechanism of olfactory memory deficits in AD. Interestingly, the dysfunctions in odor detection, recognition, and identification are early symptoms of AD progression [212,213]. The disruption of odor identification may be caused by an impairment of the MOB–anterior PC pathway, which regulates the threshold for similar odor recognition [214,215]. The alterations of the MOB-AON route, which acts as a storehouse for olfactory memory, may be a contributor to impaired odor perception [216]. The impairment of odor detection may be associated with disruption of the MOB-lateral EC pathway, which regulates olfactory coding according to odors, experiences, and states through odor-specific and restricted firing [217]. It has been established that Aβ and tau are major causes of olfactory dysfunction in AD [218,219]. Notably, impairment of the olfactory system occurs prior to AD onset. In an animal study, Tg2576 mice showed Aβ pathology in the OB before the occurrence of memory loss [220,221]. Interestingly, AD pathology in the OB may impair the olfactory system and affect several brain regions [222]. Monomeric and oligomeric Aβ from the OB propagate to other brain regions along the neural connections [222]. In summary, the olfactory system was deficient in early AD. In addition, the olfactory neuronal loss that accompanies functional impairment may provide strong evidence suggesting alterations in the olfactory pathways in AD. Despite the importance of olfactory connectivity, few studies have examined the impairment and the degree of damage in the olfactory pathways of AD models. Thus, future studies to reveal the altered olfactory pathways according to the stages of AD could provide crucial insights for the treatment of AD.

Moreover, as mentioned above, olfactory dysfunction appears earlier than AD onset; therefore, it has been recommended as a potential indicator of AD diagnosis [223]. Olfactory impairment has been proposed as a useful indicator for predicting the risk of progression from MCI to AD [224,225]. Thus, there is a growing interest in the development of diagnostic techniques that utilize olfactory impairment as an indicator of AD. For instance, the ‘left–right nostril odor detection test’, based on the asymmetrical degeneration of the olfactory pathway in AD patients, has been reported as a non-invasive and highly sensitive brief test that can help diagnose AD [226]. Thus, novel studies on AD diagnosis techniques using alteration of the olfactory pathway as an indicator are continuously being conducted [227,228].

## 3. Targeting the Neural Circuits for Treatment of AD

In this review, we identified impairments in the neural circuits of the AD brain (Figure 7). The degeneration of neural circuits is a trigger for the several clinical symptoms of AD. In particular, it is known that cognitive decline in AD is caused by the impairment of neural pathways [14,229]. Although the specific relationship between altered neural circuits and behavioral deficits in AD is not yet fully understood, it is likely that various AD-related neuropsychiatric symptoms have also been associated with neural circuit impairment [230]. Several studies have suggested that a therapeutic approach to restoring the neural circuit may be effective in improving the clinical symptoms of patients with AD [10,231]. In addition, as clinical trials targeting molecular pathologies such as Aβ and tau pathology have failed one after the other, their potential as therapeutic targets of neural circuits is increasing [9,231]. Therefore, it has been strongly suggested that the recovery of impaired neural circuits can be an effective therapeutic target for the treatment of AD symptoms, including cognitive and psychiatric deficits. Based on the accumulated evidence, we discuss the potential of neural circuits as therapeutic targets, as well as promising therapeutic approaches for neural circuits in AD treatment.

For therapeutic strategies targeting neural circuits, it is important to choose the right patient and time. The natural course of AD is as follows: preclinical, prodromal, mild, moderate, and severe AD. In addition, AD patients are categorized based on imaging and biofluid biomarkers using the ATN (A: Aβ, T: tau, N: neurodegeneration) classification system [232]. Following the framework provided by ATN classifications, the right time and patient for AD treatment targeting neural circuits would be prodromal to mild AD patients with cellular dysfunction [233]. Moreover, deficits in several neural circuits in AD occur in the early stages [13]. Strategies to restore damaged neural circuits are expected to have a high probability of success in patients with MCI. Furthermore, the combination of protecting or stimulating neural connections by targeting Aβ and tau is thought to be the optimal strategy to alleviate both the pathology and symptoms of AD. Collectively, this strategy of targeting neural circuits can be applied as a combination therapy in the early stage of AD, and can help alleviate symptoms by activating the remaining circuit in the late stage of AD.

Several potential therapeutic approaches have been proposed for the restoration of neural circuits. Optogenetics, which uses light to modulate neural circuits, is emerging as a novel approach for the treatment of CNS diseases [234]. Optogenetics has been proposed as an accurate treatment that can specifically modulate only certain types of neurons [235]. Surprisingly, the modulation of the glutamatergic pathway through optogenetic therapy in the medial PFC of rodents increased recognition memory [236]. In addition, optogenetic therapy has been shown to have a significant therapeutic effect in an AD transgenic model [235]. In Tg2567 mice, optogenetic stimulation ameliorated the decline in spatial learning and memory function by protecting the connectivity of the entorhinal–hippocampal CA1 pathway [235]. Moreover, spatial memory was improved in J20 mice via optogenetic modulation for gamma oscillations of the MS pathway [237]. Moreover, chemogenetics, which uses designer receptors exclusively activated by designer drugs (DREADDs), has also been suggested to be an effective method to modulate neural activity and correct neural circuit dysfunction [238]. Chemogenetic therapy alleviated AD pathology in 5XFAD mice and improved performance in behavioral tests by modulating the abnormal activity of neuronal pathways in TgF344-AD rats [239,240]. Therefore, viral-mediated gene therapy, including optogenetics and chemogenetics for damaged neural circuits, may be a promising treatment for AD.

Another possible approach to modulating neural circuitry to treat AD-related symptoms is through transcranial electrical stimulation (tES). tES is a non-invasive treatment that electrically stimulates the brain through the scalp and includes transcranial magnetic stimulation (TMS), as well as transcranial direct current stimulation (tDCS) [241]. TMS stimulates the brain using an intensive magnetic field [242]. In several studies, repetitive TMS restored cognitive dysfunction in patients with MCI and with mild or moderate AD [243]. Another study also suggested that TMS intervention in AD patients may contribute to the recovery of memory loss and cognitive dysfunction in the brain with AD [244]. One of the suggested potential mechanisms of TMS is the regulation of vulnerable circuit connectivity [243,244]. Based on these studies, a randomized clinical trial to verify the effectiveness of TMS in patients with AD is ongoing (NCT03121066). Furthermore, tDCS improved motor and cognitive functions, including recognition memory, in MCI and AD patients [245]. Moreover, tDCS has been suggested to enhance cognitive function in a double-blind placebo control trial in patients with mild and moderate AD [246].

Deep brain stimulation (DBS) is a surgical treatment that modulates the activation of neural circuits through a neurostimulator device placed in the brain [247]. The therapeutic effect of DBS is well known in neurodegenerative diseases [248]. In addition, accumulating evidence suggests that DBS may be an effective method for improving AD [249]. DBS for targeting the medial septum in a rat model of dementia restored spatial memory by modulating the septo-hippocampal cholinergic pathway [250]. Moreover, DBS for targeting the fornix and hypothalamus in AD patients induced the activation of memory circuits and alleviated cognitive decline [251]. Several clinical trials are underway to investigate the effectiveness of DBS in patients with MCI and AD. In addition, gamma entrainment using sensory stimuli (GENUS) improved cognitive function by mitigating AD pathology and restoring the function of neural circuits in AD mouse models [252]. In summary, there are possible therapeutic approaches that can be used to modulate neural circuits and restore damaged neural circuits in AD brains, and these therapies can be effective in improving cognitive dysfunction. However, there are still some critical questions to be considered for the effective clinical application of neural-circuit-targeted treatment of AD. Although therapeutic strategies targeting neural circuits have successfully restored the function of altered neural circuits in AD, the long-term effects of these treatments are still unknown. Moreover, the continuously increasing neuronal loss as AD progresses can reduce the effectiveness of the neural-circuit-centered approach. Thus, it is promising to discover strategies that can have neuroprotective effects in the AD brain by targeting neurodegeneration, including neuronal death, synaptic loss, and neural circuit degeneration.

## 4. Discussion

This review provided a summary of altered neural pathways at the mesoscale level (Table 1) and other levels in AD brains (Figure 7). The alteration of neural pathways, leading to cognitive decline and behavioral impairment, is an important pathology directly related to AD symptoms. Thus, we emphasize the importance of neural pathways for understanding the pathological processes and clinical symptoms of AD. Furthermore, we discussed the therapeutic implications of approaches to targeting the neural circuits in AD. Several studies and clinical trials have suggested that therapeutic methods to enhance the activity and connectivity of neural circuits are effective in ameliorating AD pathogenesis. Taken together, we conclude that strategies targeting altered neural pathways in the AD brain are potent therapeutic targets for the treatment of AD. Thus, more research is needed, to examine the alterations of the neural pathways in the brain with AD and develop the therapeutic approaches that restore or protect neural connectivity in the AD brain.

## Figures and Tables

**Table 1:** Degeneration of the efferent pathways in the brains of AD mouse models.

Regions	Models	Neural Tracers	Findings		References
Hippocampal formation	Tg2576 mouse	Fast Blue	Subiculum → NAc ↓	Decreased glutamatergic transmission from the subiculum to the NAc core.	[51]
	5XFAD mice	DiI	Hippocampal formation → MS ↓	The DG→MS and Sub→MS pathways was degenerated before cognitive decline.	[40]
Septal area	Tg601 mice	DTIDTT	MS → hippocampus ↓	The connectivity of the septo-hippocampal pathway in the old (16- to 18-month-old) mice was reduced compared to healthy and adult (six- to eight-month-old) mice.	[67]
	THY-Tau22 mice	FG	MS → hippocampus ↓	Innervation from the MS to the hippocampus decreased in the 5XFAD mice compared to WT mice.	[68]
	J20 mice	BDA	MS → hippocampus ↓	GABAergic septo-hippocampal connection was reduced in eight-month-old J20 mice compared to WT mice.	[69]
	VLW mice	BDA	MS → hippocampus ↓	The GABAergic septo-hippocampal innervation on parvalbumin-positive interneurons deteriorated in two-month-old VLW mice compared to WT mice.	[70]
	5XFAD mice	DiI	MS → hippocampus ↓	Innervation from the MS to the hippocampus decreased by about 52% in the 5XFAD mice compared to WT mice.	[12]
	5XFAD mice	BDA	MS → hippocampal formation ↓	Impairment of the connectivity of the septo-hippocampal pathway occurred before cognitive decline.	[40]
Locus coeruleus	5XFAD mice	DiI	LC → hippocampus ↓	Innervation from the LC to the hippocampus decreased by about 69.1% in the 5XFAD mice compared to WT mice.	[12]
Substantia nigra	5XFAD mice	DiI	SN → hippocampus ↓	Innervation from the SN to the hippocampus decreased by about 41.3% in the 5XFAD mice compared to WT mice.	[12]
Visual area	3xTg mice	Cholera toxin beta subunit	Retina → superior colliculus ↓	The retino-collicular pathway through which RGCs reach the terminals in the superior colliculus, which is the primary target of RGCs, is impaired in three-month-old 3xTg mice.	[71]
Olfactory area	5XFAD mice	DiI	OB → hippocampus ↓	Innervation from the OB to the hippocampus decreased by about 52% in the 5XFAD mice compared to WT mice.	[12]

Sub—subiculum; DG—dentate gyrus; DTT—diffusion tensor tractography; DTI—diffusion tensor imaging; GABA—gamma-aminobutyric acid; LC—locus coeruleus; MS—medial septum; NAc—nucleus accumbens; OB—olfactory bulb; RSg—granular division of retrosplenial cortex; SN—substantia nigra. The right arrow indicates the direction of projection, and the down arrow indicates decreased connectivity.

## Data Availability

Not applicable.
